# A novel alcohol steamed preparation from *Gastrodia elata* Blume: Pharmacological assessment of a functional food

**DOI:** 10.3389/fphar.2023.1092693

**Published:** 2023-03-22

**Authors:** Lijun Cheng, Hui Wang, Kejian Ma, Yang Deng, Maoru Li, Ji Ma

**Affiliations:** ^1^ College of Agronomy and Life Sciences, Zhao Tong University, Zhaotong, Yunnan, China; ^2^ Yunnan Gastrodia elata Green Planting and Processing Engineering Research Center, Zhaotong, Yunnan, China; ^3^ Department of Pharmacy, Huzhou Maternity and Child Healthcare Hospital, Huzhou, Zhejiang, China; ^4^ Central Laboratory, Yunnan Institute of Traditional Chinese Medicine and Materia Medica, Kunming, Yunnan, China; ^5^ School of Traditional Chinese Pharmacy, China Pharmaceutical University, Nanjing, Jiangsu, China

**Keywords:** *Gastrodia elata Blume*, alcohol steamed, anxiety, metabolomics, tryptophan metabolism

## Abstract

Rhizoma Gastrodia (Orchidaceae; *Gastrodia elata* Blume), the rhizome of *Gastrodia elata* Blume (GE), is traditionally used as both a medicinal and functional food, with proven efficacy in treating mental disorders. In traditional processing, GE is washed, steamed with water, dried, and sliced. In this study, a novel processing technology-alcohol steamed GE (AGE) was proposed as an alternative. Totally, 17 compounds were identified in fresh GE and AGE. Compared with fresh GE, the relative content of parishin A and parishin E decreased after alcohol steaming, whereas gastrodin (GAS), p-hydroxylbenzyl alcohol (HBA), Parishin B, and Parishin C were increased. Additionally, the pentobarbital-induced sleep mice model and Chronic Restraint Stress (CRS) model were applied to evaluate the pharmacological effects of fresh GE and steamed GE, and both fresh and steamed GE showed an intensive hypnotic and anti-anxiety effect. Furthermore, the anti-anxiety mechanism based on serum metabolic was investigated and the tryptophan metabolic pathway was considered the response to the anti-anxiety effect of GE. Although the optimization of the processing technology of AGE still needs to be further explored, the current results have provided new thoughts for the processing technology and clinical application of GE.

## Introduction

Mental disorder has gradually become a prominent problem worldwide, and according to a cross-sectional epidemiological study published in 2019, showed that weighted lifetime prevalence of any mental disorder was 14.6%, among them anxiety disorders (7.6%) and mood disorders (7.4%) were the most prevalence class of lifetime disorders. Depression is considered to be the second leading cause of years of disability in China (Huang et al., 2019). Although the vast majority of people are simply in a mental sub-health condition and cannot be diagnosed with depression in their daily lives, they still suffer from anxiety and sleep problems (Hongmei et al., 2014). In this instance, some functional foods may be more appropriate than specialized psychiatric drugs.

*Gastrodia elata* Blume (GE), is a kind of precious traditional Chinese medicine, mainly found in Yunnan, Sichuan, Guizhou, and Shanxi provinces, among others. GE is used to treat convulsions, epilepsy, tetanus, headaches, limb numbness, and rheumatic paralysis (Commission 2015). Modern medicine has also confirmed that GE is effective in lipid reduction, neuron protection, improving cognitive function, anti-anxiety and anti-depression (Jian et al., 2012; Liu et al., 2018; Yan et al., 2019). GE has been used in diet and medicine for thousands of years and was officially listed by the China Food and Drug Administration as a functional food material on 15 November 2021 (China 2021).

As a traditional botanical drug and food material, GE proved to be effective on depression and anxiety. Yu-En Lin et al. summarized the recent studies on the antidepressant-like effects of GE. GE and its active compounds (gastrodin, 4-hydroxybenzyl alcohol, vanillin) showed intensive effects on the depression model and the potential mechanisms including neurotransmitters, anti-oxidation, anti-inflammation, modulation of the hypothalamic-pituitary-adrenal axis, neurotrophic effects, regulation of stem cell, and enhancement of neuroplasticity and neuroprotection (Lin et al., 2018). The water extract of GE could regulate monoaminergic neurotransmission and alter gut microbiota composition and function and showed an antidepressant-like effect in a mild social defeat stress-induced depression mouse model (Huang et al., 2021). Thus, GE may be a potential healthy food for individuals with sub-optimal mental health.

GE used to be processed before clinical application for thousands of years, and the ingredient content or clinical effects would be changed as a result of processing (Ye X. et al., 2019). Historically, GE was commonly processed with yellow rice wine, refined honey, wheat bran, and even ginger juice (Yang et al., 2018), but now it is officially recommended to be steamed by using water and then dried to obtain high-quality pieces according to Chinese pharmacopoeia (Commission 2015). Our previous study indicated that more effective ingredients were extracted after water steaming, and a distinct better bio-activity was found in the water steamed GE group rather than the fresh GE group (Ma et al., 2021). Gastrodin a major component of GE, easily soluble in wine and processing GE with wine could not only fully dissolve its effective components, but also showed a better effect. It is reported that 75% ethanol extract of GE proved to be effective as a potential antidepressant drug, 7 days of oral administration of GE ethanol extract (200 mg/kg, 300 mg/kg) could significantly reduce the immobility duration in forced swimming test (FST) and tail suspension test (TST) (Zhou et al., 2006). Moreover, GE ethanol extract can improve intestinal flora health *in vitro* and provide a theoretical basis for the development of GE as a potential functional food (Fan et al., 2022). The alcohol processing technology dates back to the Tang Dynasty and has a thousand years of history in China. It is widely used in traditional botanical drug, such as Radix *et Rhizoma Rhei* [Polygonaceae, *Rheum officinale Baill.*], prepared *Rehmannia glutinosa* root [Scrophulariaceae, *Rehmannia glutinosa Libosch*] (Yang et al., 2018). Therefore, we propose a new processing method, “alcohol steaming”, which combines alcohol steaming and alcohol extraction. Alcohol steaming may be a more effective processing technique with huge potential for clinical application.

In this study, GE was processed by a new processing technology and then the changes in the effective components of GE were detected before and after alcohol steaming. In addition, the classic pentobarbital-induced sleep mice model and CRS anxiety model were established to evaluate the pharmacodynamic effect of GEs, and LC-MS-based serum metabonomic analysis was used to explore the potential mechanism of the anti-anxiety effect of fresh GE and alcohol-steamed GE. The potentially representative metabolites and associated biological pathways in the mouse brain were then examined to test the inference of serum metabolomics.

## Materials and methods

### Plant materials and chemical reagents

As described in our previous study (Ma et al., 2021), all the fresh GE samples were obtained from Xiaocaoba planting base in Zhaotong (Yunnan province, China), and were authenticated by Prof. Qin Meng (Yunnan institute of food and drug control, Kunming, China). The voucher sample (Authentication No. TM20190135) were stored at Institution of Yunnan Traditional Chinese Medicine and Materia Medica, Kunming, China. GAS, HBA, and PA (purity >99%) were purchased from National Institutes for Drug Control (Beijing, China). Methanol and acetonitrile (HPLC grade) were purchased from Tedia Company Inc. (Fairfield, United States), while the MS grade solvent was obtained from Merck Company Inc. (Darmstadt, Germany). Estazolam was purchased from Huazhong Pharmaceutical Co., LTD. (Xiangyang, China). Ultrapure water was prepared by a Milli-Q purification system (Bedford, United States). Anti-tryptophan hydroxylase 2 (TPH2), Anti-Indoleamine 1(IDO1), and Anti-Brain derived neurotrophic factor (BDNF) were purchased from Abcam Company Inc. (Cambridge, United Kingdom).

### Gastrodia elata Blume samples preparation

**FIGURE 1:**
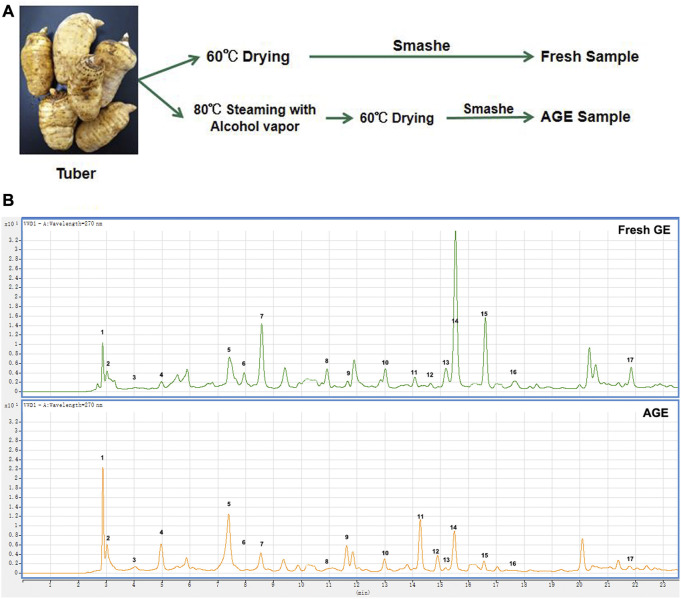
Effect of alcohol proceed on chemical ingredients in GE samples. **(A)** The preparation of fresh GE and AGE samples. **(B)** The HPLC chromatograms of fresh GE and AGE.

The fresh GE samples were driedin 60°C desiccators for 48 h. In order to prepare the alcohol-steamed GE samples, the fresh GE was firstly cooked in alcohol vapor at 80°C for 20 min and then dried at 60°C for 48 h (Figure 1A).

### Chemical ingredients detection in steaming process

The chemical ingredients of fresh GE and AGE were detected in accordance with previous studies (Li et al., 2019; Ma et al., 2021).

### Animals

All the experimental protocols on animals were approved by the Ethical Committee of China Pharmaceutical University, Nanjing, and the Laboratory Animal Management Committee of Jiangsu Province (SYXK (SU) 2018-0019). Adult male Sprague- Dawley (SD) rats (200 ± 20 g body weight) and C57BL/6 mice (20 g ± 2 g body weight) were obtained from the SLAC Laboratory Animal Co., Ltd. (Shanghai, China). During the study, all the animals were kept in a 12-h light-dark cycle at room temperature (22◦C ± 1°C) and humidity (60% ± 10%) with free access to water and a rodent chow diet.

Power analysis for sample size: The ARRIVE Guidelines (Animal Research: Reporting of *In Vivo* Experiments) were used for power analysis. We used the sleep duration index for mice experiment, and open-filed test index for rat experiment between the Model and AGE group as primary outcomes to calculate the number of animals needed in each group by “Animal Sample Size Calculations for Anticipated Means or from Pilot Study/Publication Data” (https://intraweb.hku.hk/local/launit/content/forms/Animal_Sample_Size_Calculations_for_Anticipated_Means_or_from_Pilot_Study_or_Publication_Data.xlsx). A power analysis indicated that to detect an effect size of 15 min in mean sleep duration between groups with 90% power at alpha = 0.05 in a one-way ANOVA with four levels, a sample of eight per group was necessary in the mice experiment. An effect size of 24.5 s in mean open-filed test between groups with 90% power at alpha = 0.05 in a one-way ANOVA with five levels, a sample of six per group was necessary, therefore, we used eight animals per group in rat experiment.

### Sedative and tranquilizing experiment

**FIGURE 2:**
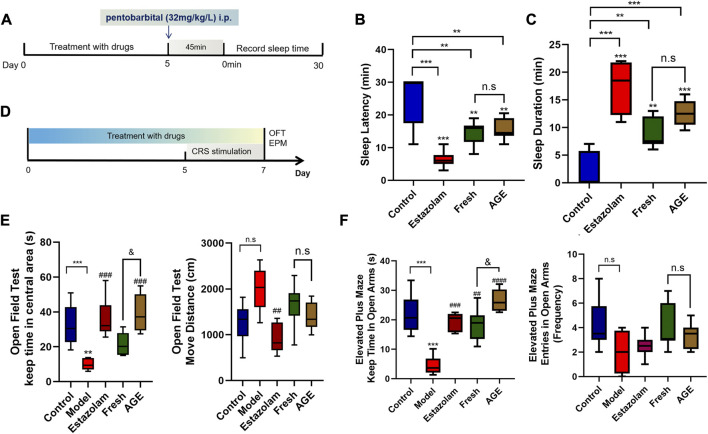
Pharmacological study effects of fresh GE and AGE. **(A)** The experiment design of pentobarbital-induced sleep mice (*n* = 8). **(B)** sleep latency time (min). **(C)** Sleep duration time (min). **(D)** The experiment design of CRS mice model (*n* = 8). **(E)** The keep time in central areas(s) and move distance (cm) in OFT test. **(F)** The keep time in open arms(s) and entries in open arms in EPM test. *p < 0.05; **p < 0.01; ***p < 0.001 represent compared with the control group. &*p* < 0.05 represent compared with fresh GE and AGE groups. *p* < 0.05 was considered statistically significant calculated by One-way ANOVA followed by Tukey’s test.

The phenobarbital-induce sleep mice model was performed according to our previous study (Ma et al., 2021). The rats were randomly divided into four groups: control, estazolam, fresh GE, and alcohol-steamed GE (AGE). The mice were respectively intraperitoneally injected with an equal volume of 0.5% CMC-Na solution, estazolam (1 mg/kg), fresh GE (1.1 g/kg), and AGE (1.1 g/kg) for 5 days. Then, a sub-hypnotic dose of phenobarbital (32 mg/kg/L) was administrated *via* peritoneal injection. The sleep latency and sleep duration time were observed and recorded after 45 min later (Figure 2A).

### Evaluation of the anxiolytic effect

Previous studies have demonstrated that both fresh GE and water-steamed GE have anti-anxiety-like activity. To further compare the anxiolytic effect of AGE, 40 animals were divided into five groups (8 mice per group): control, model, estazolam, fresh GE, and AGE. At the beginning of the experiment, all animals were accordingly treated with an equal volume of 0.5% CMC-Na solution, estazolam (1 mg/kg), fresh GE (1.1 g/kg), and AGE (1.1 g/kg) by gavage for 7 days. The chronic restraint stress model was established as described previously (Ma et al., 2021), and the group without CRS stimulation was set as a control. The open field test (OFT) and elevated plus maze test (EPM) were used to assess the anxiety behavior of animals on the last day (Figure 2D).

### Serum non-targeted metabonomic analysis on anxiety animals

All animals were sacrificed after the behavioral test and their serum and whole brain tissue were collected for stepping detection. Serum samples (eight samples per group) were prepared and detected by the method mentioned in our previous study (Ma et al., 2021).

Non-targeted-metabonomic was carried out on a 1260 HPLC system coupled to 6500 quadrupoles of flight (Q-TOF) MS (Agilent, Santa Clara, CA) with a poroshell 120 SB-C18 column (2.1 × 100 mm, 2.7 µm). A flow rate of 0.4 mL/min was used, and the injection volume was 5 μL. The mobile phase consisted of A (water with 0.1% formic acid) and B (acetonitrile) in the positive mode analysis, while A (1 mM ammonium fluoride in the water) and B (acetonitrile) in the negative mode analysis. The following gradient elution conditions were used: 0–2 min, 2% B; 2–20 min, 2%–100% B; 20–25 min, 100% B.

The electrospray ionization (ESI) source was operated both in positive and negative ion modes and MS parameters were optimized as follows: Gas Temp, 325°C; Gas Flow, 8 mL/min; Nebulizer, 40 psig; Sheath Gas Temp, 350°C; Sheath Gas Flow, 12 mL/min; V Cap, 4000; Nozzle Voltage, 0 V (positive mode), and 500 V (negative mode); Fragmentor, 150 V; Skimmer, 65; Octopole RF peak, 750. And mass scan range, m/z 50–1000.

Additionally, Amixture of each sample was considered as the quality control (QC) sample to evaluate instrumental precision, and the QC sample was detected after every eight samples during the whole sample batch.

Principal component analysis (PCA) and orthogonal partial least squares-discriminant analysis (OPLS-DA) was performed using the online MetaboAnalyst platform (https://www.metaboanalyst.ca). Ions with a VIP >1 and a significant *t*-test result (*p* < 0.05) were selected for identification. The HMDB database was used to match ions, and the KEGG database was used for pathway analysis after the secondary identification of matched ions by LC-MS/MS.

### The relative content of tryptophan-related metabolites in brain tissue samples

The brain tissue samples (50 mg) were homogenized with 1 mL 4°C precooling methanol for 15 min, then vortexed for 3 min to precipitate the protein. The samples were then centrifugated at 14,800 g for 15 min at 4°C. The supernatant was transferred to a clean EP tube (1.5 mL) and dried. Residues were reconstituted in 50 μL pre-cooled acetonitrile aqueous (50%) and continued to be centrifugated at 14,000 rpm for 15 min at 4°C. Finally, the supernatant was then transferred into the vials for the stepping analysis. The relative content of L-tryptophan, serotonin, kynurenine, kynurenic acid, and Quinolinic acid was detected on LC/MS standard reference material purchased from Sigma.

### Detection of brain TPH2, IDO1, and BDNF protein expression by Western Bot

The total protein in the brain tissue was extracted using the RIPA lysis buffer, and protein concentrations determination was performed using a Pierce BCA protein assay kit. Subsequently, the samples were separated by 10% SDS-PAGE electrophoresis (Ye P. et al., 2019). The following antibodies were used: anti-TPH2 (1:1000), anti-IDO1 (1:1000), and anti-BDNF (1:1000). Protein expression difference was quantified using Image J Software and normalized to the expression of GAPDH.

### Data analysis

Data were represented as the mean ± SD. Statistical analysis of the results was determined using one-way ANOVA with Tukey’s correction for multiple comparisons by GraphPad Prism Software (United States). A value of *p* < 0.05 was considered statistically significant. Non-targeted metabolomics data analysis was processed following our previous study (Ma et al., 2021).

## Results

### Effect of alcohol proceeds on chemical ingredients in RGE

A total of 17 compounds were identified in fresh GE and AGE (Figure 1B) and compared with fresh GE, the relative content of Parishin A (PA) and Parishin E (PE) decreased significantly after alcohol steaming, whereas Gastrodin (GAS), P-hydroxy benzyl alcohol (HBA), Parishin B (PB), and Parishin C (PC) were increased gradually (Table 1). The alcohol steaming process was shown to effectively separate the active components from GE, which is consistent with previous research on water-steamed GE (Li et al., 2019; Ma et al., 2021). Notably, the relative content of GAS, PA, and HBA changed more significantly after alcohol steaming.

**TABLE 1:** The relative contents of 17 active ingredients in fresh GE and AGE samples.

Peak no.	Retention Time/min	Compound	Molecular formula	M	Fragment peaks	Fresh GE	AGE
1	2.86	GLUTAMIC ACID	C5H9NO4	147.0532	148.0617 [M + H]+	1	2.219
2	3.01	SUCROSE	C12H22O11	342.1162	365.1070 [M + Na]+	1	1.231
3	4.47	L-PYROGLUTAMIC ACID	C5H7NO3	129.0426	130.0500 [M + H]+	1	1.291
4	4.8	CITRIC ACID	C6H8O7	192.027	214.9170 [M + Na]+	1	2.875
5	7.44	GASTRODIN	C13H18O7	286.1052	309.0951 [M + Na]+	1 (300 mg/g)	4.141 (1242 mg/g)
6	7.55	GASTRODIN A	C19H28O12	448.1581	471.1473 [M + Na]+	1	0.055
7	8.63	ADENOSINE	C10H13N5O4	267.2413	268.1056 [M + H]+	1	0.425
8	11.12	PARISHIN E	C19H24O13	460.1217	483.1126 [M + Na]+	1	0.257
9	11.71	P-HYDROXYBENZYL ALCOHOL	C7H8O2	124.0524	125.0511 [M + H]+	1 (1120 mg/g)	6.109 (6832 mg/g)
10	13.07	S-(4-HYDROXYBENZYL) GLUTATHION	C17H23N3O7S	413.4454	414.1400 [M + H]+	1	0.786
11	14.34	PARISHIN B	C32H40O19	728.2164	751.2075 [M + Na]+	1	12.527
12	14.92	PARISHIN C	C32H40O19	728.2164	751.2050 [M + Na]+	1	16.208
13	15.28	PARISHIN K	C33H42O19	742.6752	765.2253 [M + Na]+	1	0.426
14	15.56	PARISHIN A	C45H56O25	996.9111	1019.3040 [M + Na]+	1 (1220 mg/g)	0.365 (450 mg/g)
15	16.44	PARISHIN L	C46H58O26	1026.3216	1049.3036 [M + Na]+	1	0.123
16	17.75	PARISHIN W	C26H30O14	566.1636	589.1544 [M + Na]+	1	0.005
17	21.93	PARISHIN D	C20H20O9	404.1107	427.1007 [M + Na]+	1	0.321

### Sedative and tranquilizing effects of fresh GE and AGE

Compared with the control group, both fresh GE and AGE could significantly shorten the sleep latency time and prolong the duration of sleep, while there was no marked difference between them (Figures 2B, C).

### Anxiolytic effect of fresh GE and AGE

OFT and EPM were commonly used to evaluate the locomotion and exploration of animals, which were applied to assess the anti-anxiety effect of animals. Interestingly, we found that the central mobile time of OFT and the keep time in open arms of EMP significantly increased in both fresh GE and AGE groups, among them the effects of AGE even nearly to positive group (Figures 2E, F). It was observed that AGE showed a strongeranxiolytic effect than fresh GE.

### Comparison of serum metabonomic change in anxiety mice

**FIGURE 3:**
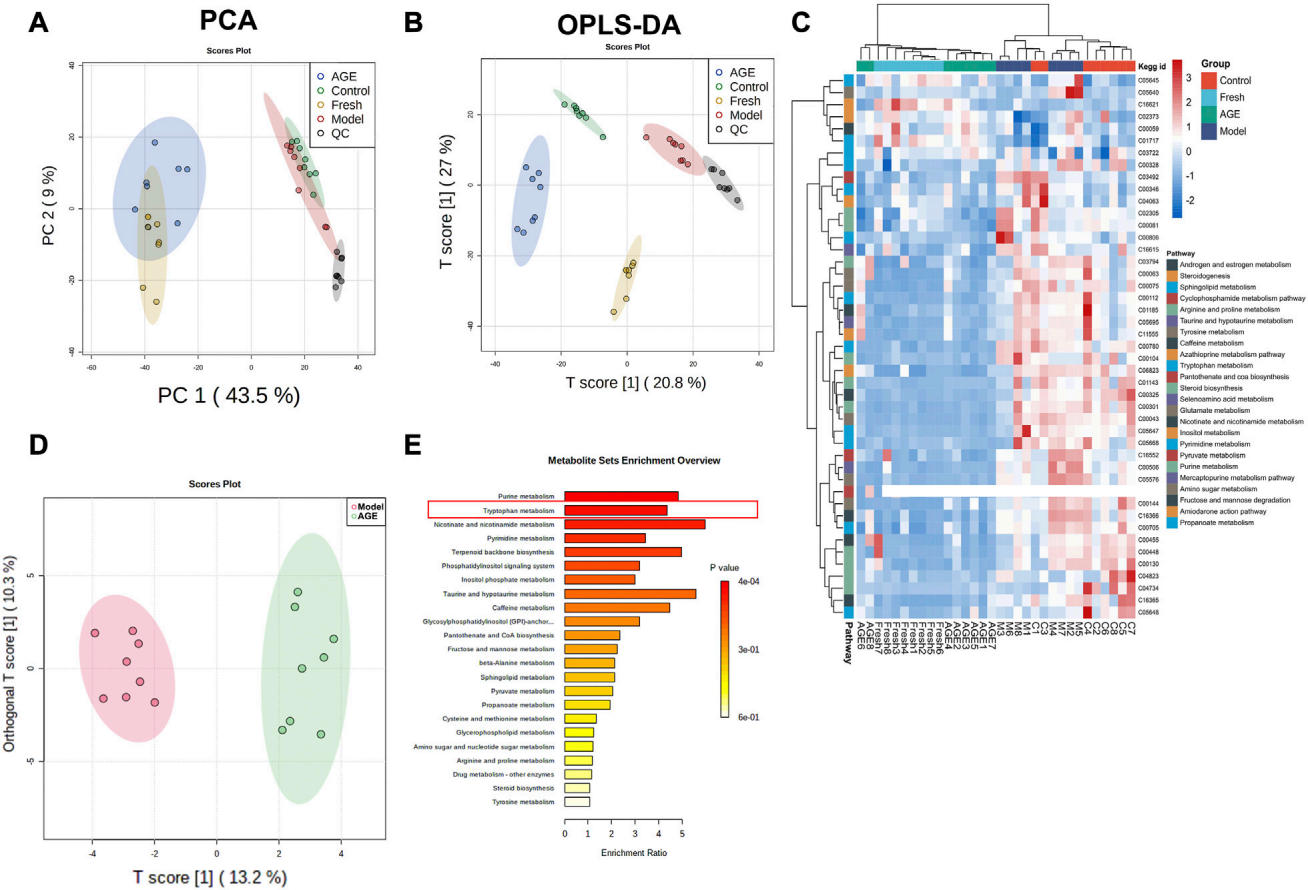
Comparison of serum metabonomic change between fresh GE and AGE in anxiety mice (*n* = 8). **(A)** The score plots of principal component analysis (PCA) scores for serum samples, **(B)** OPLS-DA analysis for serum samples, **(C)** the heatmap comparison of metabolites in serum samples, **(D)** OPLS-DA score plots of Model and AGE group metabolites, **(E)** metabolic pathway enrich map in serum samples.

Non-targeted metabolomics analysis was performed on the serum samples in fresh GE and AGE-treated groups, and a total of 2119 ions were obtained by non-targeted metabolic profiling in ESI positive and negative modes. Firstly, an unsupervised principal components analysis (PCA) was conducted for dimensionality reduction analysis, the result showed that the fresh GE and AGE groups overlap each other but distinguished from the other two groups (Figure 3A), which indicated that both fresh GE and AGE affect serum metabolic profiling. Furthermore, supervised OPLS-DA analysis was applied to find the differential variables between groups (Figure 3B). The OPLS-DA results showed that four groups could be well distinguished in serum samples. Both the fresh GE and AGE groups were close to the control group, which indicated fresh GE and AGE can ameliorate the abnormal metabolic changes in the model group, among them AGE group was more effective, which is consistent with previous anti-anxiety behavior test results.

The metabolites with a VIP value of OPLS-DA >1 and *p*-value < 0.05 will be regarded as important differential metabolites between the control and the model group. A total of 46 ions were identified as potential biomarkers, and hierarchical cluster analysis of these potential biomarkers in plasma showed that the metabolites in two drug-treated groups were separated from the control and model groups (Figure 3C). These results indicated that both fresh GE and AGE have intervention effects on anxiety in the mice.

Moreover, OPLS-DA analysis was applied to find the differential variables between AGE and model groups (Figure 3D), and these significant metabolites were imported into MetaboAnalyst 5.0 for pathway enrichment (Figure 3E). The pathway with a *p*-value < 0.05 was considered the potential target pathway associated with the anti-anxiety effect of AGE. Interestingly, we noticed that the TOP2 pathway was the mood disorder-related tryptophan metabolism pathway, which should be further investigated.

### Comparison of tryptophan-related metabolites in whole brain samples

**FIGURE 4:**
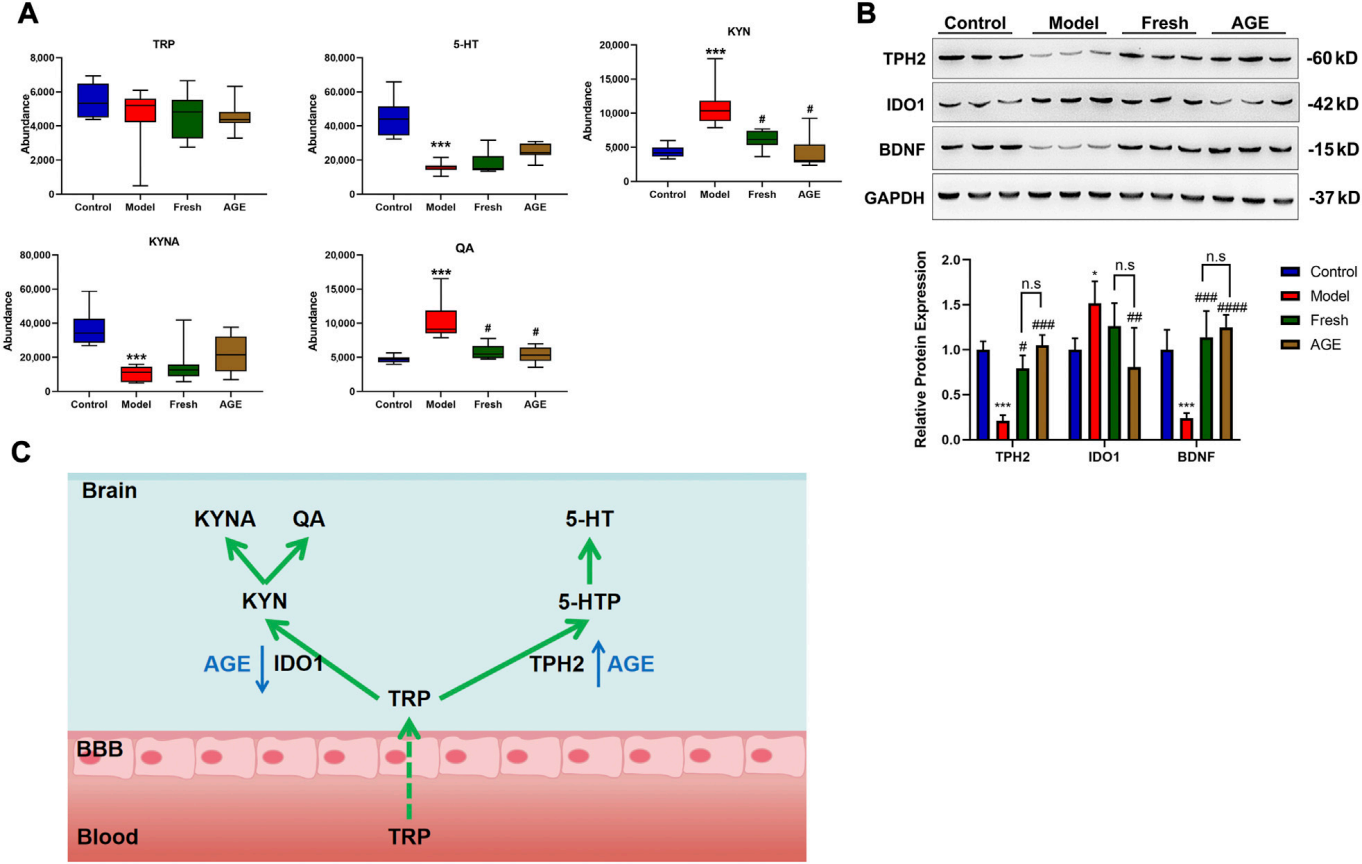
The effect of fresh GE and AGE intervention on the tryptophan metabolism in whole brain samples. **(A)** Tryptophan related metabolites, **(B)** the rate-limiting enzyme of tryptophan metabolic pathway. **(C)** Integration analysis of the AGE target pathways in anxiety mice. *p < 0.05; **p < 0.01; ***p < 0.001 represent compared with the control group. #*p* < 0.05; ##*p* < 0.01; ###*p* < 0.001 represent compared with the model group. ns represents no significant difference between fresh GE and AGE groups. *p* < 0.05 was considered statistically significant calculated by One-way ANOVA followed by Tukey’s test. TRP (tryptophan), 5-HT (serotonin), KYN (kynurenine), KYNA (kynurenic acid), QA (quinolinic acid). IDO1 (indoleamine2,3-dioxygenase1) TPH2(Tryptophan hydroxylase 2 enzyme).

According to the enriched metabolic pathway, tryptophan metabolism might be the potential metabolic pathway that is related to psychological diseases, as mentioned in the previous study (Roager and Licht 2018). We further detected the relative content change of tryptophan-related metabolites in whole brain samples by LC-MS. As expected, CRS significantly decreased the brain 5-HT and KYNA levels while increasing the QA and KYN levels, which were improved by AGE intervention (Figure 4A).

### TPH2, IDO1, and BDNF protein expression in whole brain samples

To validate the tryptophan-related metabolites changes, we detected the key enzyme for the tryptophan metabolic pathway in the brain (IDO1 and TPH2) by western blot. The expression of TPH2 significantly decreased in the CRS model group while both fresh GE and AGE could significantly improve the expression (Figure 4B). In contrast, IDO1 significantly increased in the model group and only AGE could reduce IDO1 expression induced by CRS stimulation. Moreover, BDNF is known to modulate behavioral stress response and to mediate the therapeutic effects of antidepressant agents, which interact with the 5-HT system at several levels (Homberg et al., 2014). We found that BDNF significantly decreased in the CRS group, while improved by fresh GE and AGE.

## Discussion

GAS and HBA are regarded as the quality evaluation indicators of GE in Chinese Pharmacopoeia (2015 version). Furthermore, the determination of characteristic chromatograms of PA, PB, PC, and PE was added to the Chinese Pharmacopoeia (2020 version). Among them, the content of GAS is affected by various factors and the GAS content varies greatly under different temperature and solvent conditions (Sha et al., 2012). In this study, 17 active ingredients were identified according to the mass spectrometer, and the relative content of GAS, HBA, and some smaller molecular compounds was increased in AGE, while the higher molecular parishins were decreased in AGE. It is reported that steaming process could promote the chemical transformation of active ingredients such as parishins and inhibit the enzymatic degradation of GAS (Li et al., 2019). The results further verify this conclusion and the relative content of active ingredients changed more obviously after alcohol steaming process compared with our previous research on water steamed GE (Ma et al., 2021).

Modern studies have indicated that GE and its active ingredients have an intensive antidepressant effect *in vivo* and *in vitro* (Lin et al., 2018). The OFT test was used to evaluate the spontaneous activity and exploratory behavior which reflected the anxiety degree of the mice. The EPM test was recommend to assessing the anxiety behavior on rodent models (O'Connor 2016). Thus, phenobarbital induced sleep mice model was applied to evaluate the hypnotic effect while OFT and EPM tests were used to assess the anti-anxiety effect in this article. Both fresh GE and AGE have a well hypnotic effect (Figures 2B, C), whereas AGE showed better effects in the anti-anxiety test compared with fresh GE (Figures 2E, F).

In order to have a deeper understanding of the GE potential anti-anxiety mechanism, we constructed a serum metabolomic analysis to evaluate the metabolic profile changes in different groups. The results revealed that both fresh GE and AGE could reverse the metabolic disorder induced by CRS (Figures 3A–C). Moreover, we compared the differential metabolites among the model and AGE groups (Figures 3D, E). Among these s differential metabolic pathways, tryptophan metabolism was reported to have key roles in varied neuropsychiatric diseases and is closely related to mood regulation (Curzon 1978; Roager and Licht 2018). Therefore, we proposed that the tryptophan metabolic pathway may be involved in anti-anxiety effects in response to AGE.

In general, majority of tryptophan is metabolized in the body through the kynurenine pathway (95%) and the serotonin pathway (2%), while others are metabolized through the gut microbiome (Agus et al., 2018). TRP-KYN pathway is mediated by the rate-limiting enzyme Indolamine 2,3-dioxygenase (IDO1) and leads to the production of kynurenine and downstream products such as neuroprotective kynurenic acid (KYNA) and neurotoxicity quinolinic acid (QA) (Platten et al., 2019). Moreover, as an important monoamine neurotransmitter, 5-HT is produced through the rate-limiting enzyme Tryptophan hydroxylase 2 enzyme (TpH2) in the brain, and abnormalities in different processes of serotonergic activity might initiate depression. On this basis, the antidepressant agents 5-HT reuptake inhibitors (SSRIs) and tricyclic antidepressants (TCAs) have been developed and applied for the treatment of depression (Dell’Osso et al., 2016).

In this study, compared with the control group, 5-HT and KYNA levels significantly decreased, while KYNA and QA increased in the model group (Figure 4A), Meanwhile, the expression of TPH2 significantly decreased and IDO1 increased (Figure 4B). The results claimed the limitation of the tryptophan-5-HT pathway and the overactive of the tryptophan-KYN pathway, which might be one of the mechanisms leading to anxiety-like behavior in mice (Curzon 1978). Both fresh GE and AGE could reverse the metabolic disorder in the tryptophan pathway induced by CRS, but only AGE significantly reduces IDO1 expression induced by CRS stimulation.

In addition, BDNF, the most abundant neurotrophic factor, is generally known to play a key role in neurogenesis and synaptic sprouting and is modulated by antidepressant agents as a down-stream target of 5-HT (Homberg et al., 2014). The expression of BDNF was found to be decreased in the hippocampus of patients with depression and the upregulation of BDNF might be used in the antidepressant treatment (Duman and Monteggia 2006). We found that BDNF significantly decreased in the CRS group which was improved by fresh GE and AGE intervention (Figure 4B). Our results provide an anti-anxiety potential of fresh GE and AGE, tryptophan related metabolic pathways might be the possible causes of the better effects in the anti-anxiety test of AGE **(** Figure 4C **)**.

Finally, this study is still in the earlystages of exploring the effects of AGE Further research is needed to investigate the, the pharmacology study of the AGE extraction or the combination of active compounds, as well as the dose range and toxicity study of the alcohol-steamed preparation method. This study provides valuable insights and encourages future studies on the potential benefits of AGE.

## Conclusion

A novel processing technology, alcohol steamed GE, was proposed. The chemical changes and pharmacologic effects before and after the steaming process were investigated. Interestingly, it was found that AGE is efficient in inducing hypnotic and anti-anxiety effects, as evidenced by behavior tests and serum metabonomic analysis. The difference in the tryptophan-related metabolic pathway was observed and may be the possible cause of AGE’s improved pharmacologic efficiency. However, the study has limitations, such as the need for further optimization of the processing method and the absence of metabolomic analysis in specific brain regions. In the future, the optimization of the AGE processing technology and its detailed pharmacologic mechanism will be further explored.

## Data Availability

The original contributions presented in the study are included in the article/supplementary material, further inquiries can be directed to the corresponding author.
